# Furin cleavage of SARS-CoV-2 Spike promotes but is not essential for infection and cell-cell fusion

**DOI:** 10.1371/journal.ppat.1009246

**Published:** 2021-01-25

**Authors:** Guido Papa, Donna L. Mallery, Anna Albecka, Lawrence G. Welch, Jérôme Cattin-Ortolá, Jakub Luptak, David Paul, Harvey T. McMahon, Ian G. Goodfellow, Andrew Carter, Sean Munro, Leo C. James

**Affiliations:** 1 MRC Laboratory of Molecular Biology, Francis Crick Avenue, Cambridge, United Kingdom; 2 Division of Virology, Department of Pathology, University of Cambridge, Addenbrooke’s Hospital, Hills Road, Cambridge, United Kingdom; Icahn School of Medicine at Mount Sinai, UNITED STATES

## Abstract

Severe Acute Respiratory Syndrome coronavirus 2 (SARS-CoV-2) infects cells by binding to the host cell receptor ACE2 and undergoing virus-host membrane fusion. Fusion is triggered by the protease TMPRSS2, which processes the viral Spike (S) protein to reveal the fusion peptide. SARS-CoV-2 has evolved a multibasic site at the S1-S2 boundary, which is thought to be cleaved by furin in order to prime S protein for TMPRSS2 processing. Here we show that CRISPR-Cas9 knockout of furin reduces, but does not prevent, the production of infectious SARS-CoV-2 virus. Comparing S processing in furin knockout cells to multibasic site mutants reveals that while loss of furin substantially reduces S1-S2 cleavage it does not prevent it. SARS-CoV-2 S protein also mediates cell-cell fusion, potentially allowing virus to spread virion-independently. We show that loss of furin in either donor or acceptor cells reduces, but does not prevent, TMPRSS2-dependent cell-cell fusion, unlike mutation of the multibasic site that completely prevents syncytia formation. Our results show that while furin promotes both SARS-CoV-2 infectivity and cell-cell spread it is not essential, suggesting furin inhibitors may reduce but not abolish viral spread.

## Author summary

The main focus of research into the COVID-19 pandemic is the SARS-CoV-2 Spike (S) protein, which is the viral protein responsible for binding the ACE2 receptor on the host cell. Prior to and after attachment, the S needs to be activated by cellular proteases (e.g. furin, TMPRSS2), triggering the virus entry into the target cell. The unique feature of SARS-CoV-2 S is the presence of a multibasic site, an amino acid motif recognised by furin protease, and whose cleavage activates the S protein. Intensive antiviral research has been focused on developing furin inhibitors against SARS-CoV-2 infection. Here we show that furin is not absolutely required for SARS-CoV-2 virus production, suggesting that current SARS-CoV-2 antiviral therapies based on furin-targeting drugs may not completely prevent viral infection. Apart from the virus entry process, the S protein plays a key role in triggering cell-cell fusion, potentially allowing the quick spread of the virus among neighbouring cells. We show that furin-mediated pre-activation of S protein in virus infected cells is not necessary for triggering cell-cell fusion, while the multibasic site and the concomitant presence of TMPRSS2 protease on host cell membrane plays an important role in orchestrating the formation of multinucleated cells. Our results also suggest a key role of TMPRSS2 protease in promoting S-mediated cell-cell fusion paving the way for utilising a cocktail of inhibitors to efficiently treat SARS-CoV-2 infections.

## Introduction

In late 2019, a new coronavirus was identified in the Chinese province, Hubei, and named Severe Acute Respiratory Syndrome Coronavirus 2 (SARS-CoV-2) for its close similarity to severe acute respiratory syndrome (SARS-CoV) that appeared in 2002. SARS-CoV-2 spread rapidly, so that the World Health Organisation had to declare a pandemic on March 11^th^. By 3^rd^ December, it had infected more than 65 million people and caused more than 1.5 million deaths worldwide. Several studies have shown that the introduction of the SARS-CoV-2 into humans was the result of zoonotic transmission, with pangolins acting as an intermediary host between bats and humans [1,2].

SARS-CoV-2 entry into host cells is mediated by the spike (S) protein, which is a major surface protein incorporated into the viral envelope [3]. S protein is a trimeric transmembrane glycoprotein whose ectodomain includes two main subunits: S1, responsible for attachment to the host cell receptor Angiotensin-Converting Enzyme 2 (ACE2) and for shielding the S2 subunit that contains the fusion machinery [4]. Similar to the fusion proteins of many other respiratory viruses, S protein of SARS-CoV-2 is activated by cellular protease-mediated cleavage [5–8]. Activation of S requires proteolytic cleavage at two distinct sites: in the unique multi-basic site motif of Arg-Arg-Ala-Arg (RRAR), located between the S1 and S2 subunits, and within the S2 subunit (S2’) located immediately upstream of the hydrophobic fusion peptide that is responsible for triggering virus-cell membrane fusion [9–11].

SARS-CoV-2 S is a type I transmembrane protein which, besides being incorporated into virions and mediating viral entry, is present at the plasma membrane of infected cells. Plasma membrane-displayed S protein can trigger fusion with neighbouring cells, leading to the formation of multinucleate enlarged cells (termed syncytia) [12–14]. This event is dependent upon expression of the cell-surface serine protease TMPRSS2, which exposes the S fusion peptide and makes S protein fusogenic directly at the host cell membrane [12]. These syncytia have also been found in tissues from individuals infected with SARS-CoV-2, suggesting an involvement in pathogenesis [15–17].

One significant difference between S protein of SARS-CoV-2 and SARS-CoV is that only the former contains a multi-basic cleavage site [9,13]. The acquisition of a multibasic site by insertion of basic amino acids into surface glycoproteins has long been known to be a central virulence factor for many viruses including highly pathogenic influenza viruses [18–23]. This may be due to the fact that viruses with a multibasic site are readily cleaved by ubiquitously expressed proprotein convertases and can support systemic spread of infection, as showed in mammals and poultry [19,20,24,25]. In the case of SARS-CoV-2 virus, inhibitor experiments have suggested that the protease responsible for cleaving S protein is furin [13,26]. Furin is a calcium-dependent protease that recognises and cleaves the specific sequence motif R-X-R/K-R, where X can be any amino acid residue [27,28]. Furin is mainly expressed in the trans-Golgi network with little some present in other intracellular vesicles, suggesting that it may act on S protein during viral production [13,29]. In the context of SARS-CoV-2, the acquisition of a multibasic site in the S protein has been identified as a determinant of pandemic potential but its precise role in virus transmission remains unclear [30,31]. Here we sought to determine whether furin is essential for SARS-CoV-2 S multibasic site processing and how it impacts both viral entry and cell-cell spread. We find that although loss of furin markedly reduces infection it does not totally abolish it. Thus, although furin is a highly important cofactor, it is not absolutely essential for SARS-CoV-2 infection and replication will occur in its absence.

## Results

### SARS-CoV-2 S protein mediates cell-cell fusion between different cell types

**Fig 1:**
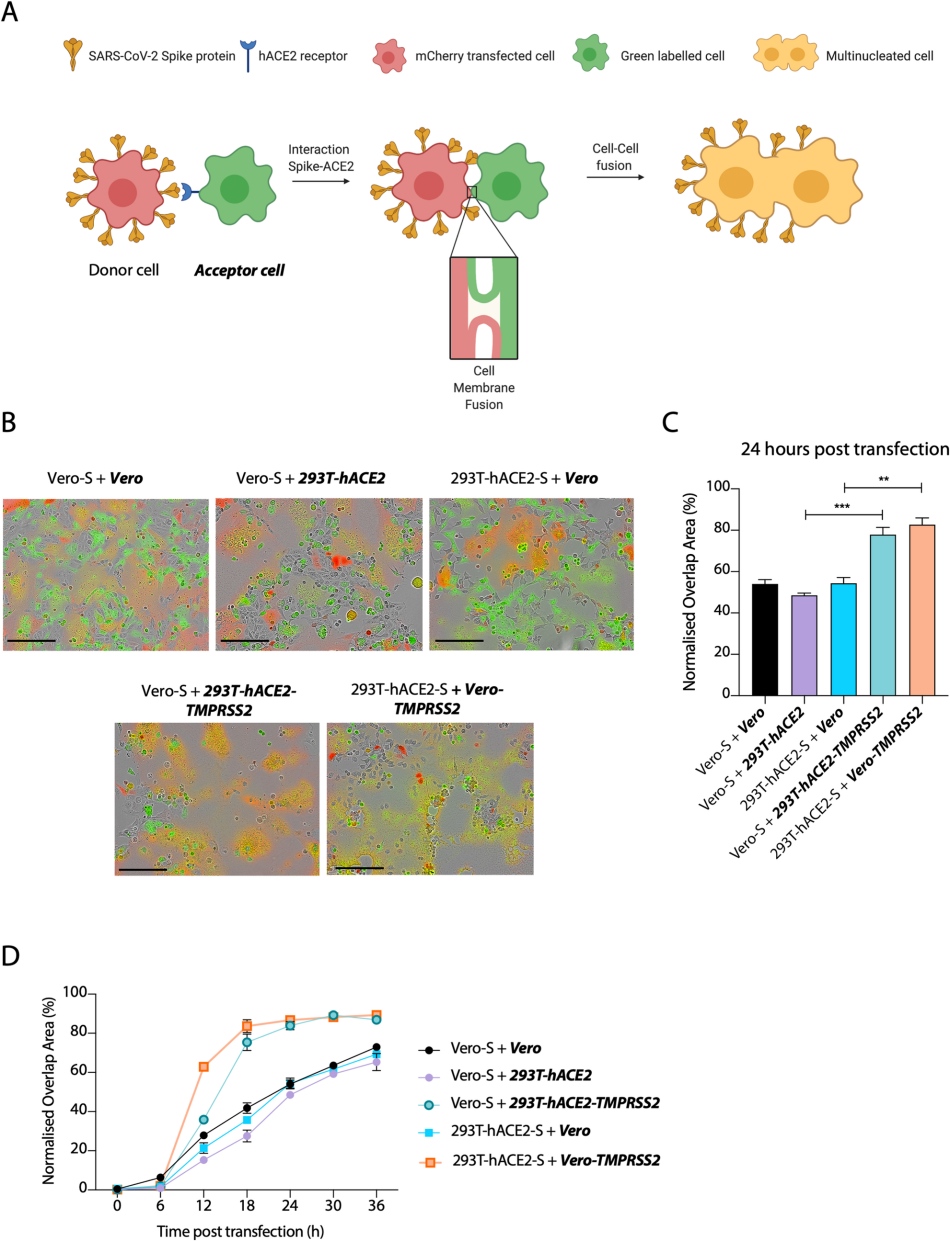
SARS-CoV-2 S protein mediates cell-cell fusion between different cell types. **(A)** Schematic representation of S protein-mediated cell–cell fusion assay. The donor cell is identified as the cell co-expressing SARS-CoV-2 S and mCherry while the acceptor cell is green-labelled. The scheme was created with BioRender.com. **(B)** Merged images at 24 hours post transfection of the indicated cell lines transfected with SARS-CoV-2 S and mixed with green dye-labelled cells. Scale bars represent 200 μm. Green colour identifies the acceptor cells while red colour marks donor cells. Merged green-red colours indicate the syncytia. **(C)** Quantification of (B) showing percentage of green and red overlap area at 24 hours post transfection. Statistical analysis was performed using Student *t*-test. *** *P<*0.001; ** *P<*0.01 **(D)** Quantification of the cell-cell fusion kinetics shown in (B). Acceptor cells are marked in **bold** and *italics*.

Previous reports have shown that SARS-CoV-2 S protein possesses high fusogenic activity and is able to trigger large syncytia formation *in vitro* and *in vivo*, contrary to the S protein of two related coronaviruses SARS-CoV and MERS-CoV [13,15]. Whether cell-cell fusion and formation of syncytia can occur between uninfected and infected cells and if it can happen between different cell types are significant questions in understanding SARS-CoV-2 pathogenesis. To address these questions, we established a SARS-CoV-2 S protein-mediated cell–cell fusion assay employing 293T cells stably overexpressing human ACE2 receptor (herein named 293T-hACE2) (S1A Fig) and Vero cells, as both have been widely used in several coronavirus studies [32]. Our assay is based on the ectopic overexpression of SARS-CoV-2 S together with the mCherry fluorescent protein in one cell type (named the donor cell) and the labelling of another cell type (named the ***acceptor* cell**) with a green fluorescent dye (Fig 1A). Mixing both cell types and measuring the kinetics of merged fluorescence allows precise quantification of cell-cell fusion. Using this assay, we investigated the fusogenic properties of S protein when present at the cell surface [33]. We found that SARS-CoV-2 S-mediated syncytia formation occurred regardless of whether Vero or 293Ts were used as donor or acceptor cells (Fig 1B and 1C). Moreover, we observed similar kinetics of syncytia formation whether we used Vero cells as both donor or acceptor or just one or the other. Next, we compared the impact of hACE2 overexpression with that of the protease TMPRSS2, which is responsible for cleavage and exposure of the fusion peptide prior to fusion [4,34,35]. Importantly, overexpression of the TMPRSS2 protease in either Vero or 293T cells, markedly increased the rate of cell-cell fusion (Figs 1B, 1C and 1D and S1B). When TMPRSS2 was overexpressed on acceptor cells, syncytia formation reached almost 85% within 24 hours post transfection (Fig 1D). This suggests that TMPRSS2 may be rate limiting for cell-cell fusion and indicates its importance in SARS-CoV-2 spread. The data also suggest that the fusion machinery of SARS-CoV-2 is an important target for development of coronavirus antivirals.

### Furin protease is not essential for the cleavage of S protein but enhances its processing

**Fig 2:**
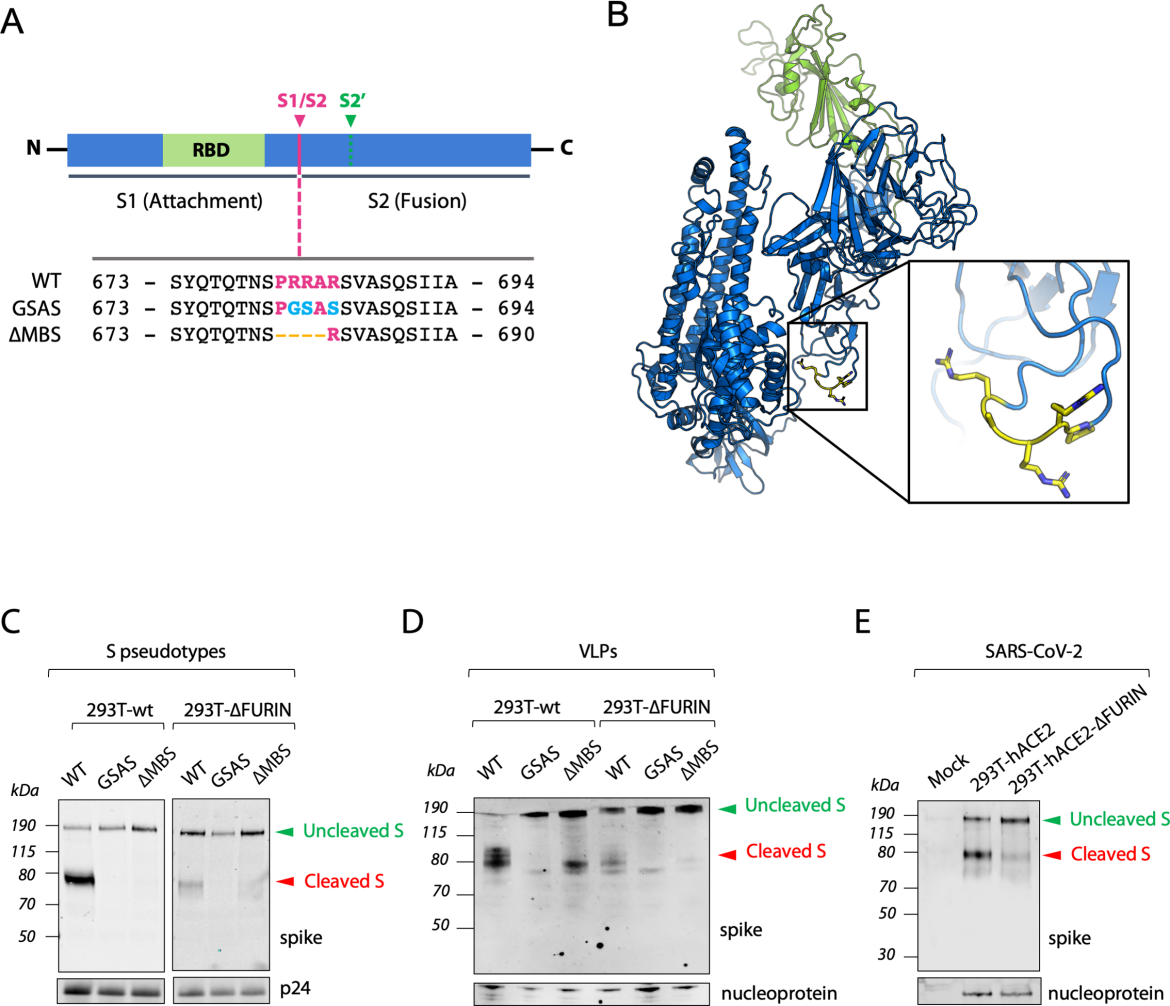
Furin is not essential for the cleavage of S protein but enhances its processing. (**A)** Schematic illustration of SARS-CoV-2 S including receptor binding domain (RBD) in green and proteolytic cleavage sites (S1/S2, S2’). Amino acid sequences around the S1/S2 recognition sites of SARS-CoV-2 S are indicated while the multibasic site is highlighted in purple. Amino acid mutations are highlighted in light blue while deletions are marked with orange dashes. **(B)** Overall structure of the SARS-CoV-2 S protein (PDB: 6VYB). RBD core is shown in green. Pro-Arg-Arg-Ala-Arg residues are shown in yellow. **(C,D)** Representative western blots of HIV Pseudoviruses **(C)** and Virus Like Particles (VLPs) **(D)** harbouring the indicated SARS-CoV-2 S protein mutants (detected with anti-S antibody) and produced in 293-wt and 293T-ΔFURIN cells. Expression of HIV capsid protein (p24) (C) and SARS-CoV-2 nucleoprotein (D) is shown as loading control. **(E)** Representative western blot analysis of spike and nucleoprotein present in SARS-CoV-2 viral particles produced in 293T-hACE2 and 293T-hACE2-ΔFURIN after 42 hours post infection. The cleaved S in (C) (D) and (E) identifies the S2 subunit.

Furin is widely agreed to be the protease responsible for cleavage of the SARS-CoV-2 S protein at the multibasic site. However, most studies have relied on small molecule inhibition or mRNA depletion of furin [3,13,36]. We decided to investigate the importance of furin by generating a 293T CRISPR-derived knockout cell line (herein named 293T-ΔFURIN). We confirmed that FURIN gene had been knocked out by Western blotting and, as shown in S1C Fig, there is no detectable furin protein in the knockout cells. We also generated different versions of the SARS-CoV-2 S protein harbouring amino acid substitutions in the multibasic site RRAR–either a complete inactivation by mutation to GSAS (GSAS) or a RAR amino acid deletion (ΔMBS), resembling the S protein sequence present in SARS-CoV [13,37] (Fig 2A and 2B). We first analysed the role of furin and multibasic site in S protein processing by producing lentiviruses pseudotyped with the SARS-CoV-2 S protein in both 293T-ΔFURIN cells and 293T parental cell line and analysing S that had been incorporated into viral particles. While wild-type SARS-CoV-2 S displayed on pseudovirions produced in parental 293T cells was 80% cleaved, S protein cleavage was strongly diminished but not absent when particles were generated in the 293T-ΔFURIN cell line. This indicates that S protein processing can happen independent of furin, but that the presence of the protease strongly enhances cleavage (Fig 2C). In contrast, pseudoviruses harbouring the GSAS or ΔMBS mutants did not show any S cleavage when produced in either cell lines (Fig 2C).

We next wanted to investigate the impact of these findings on the formation of SARS-CoV-2 virus-like particles (VLPs). In contrast to pseudotyped viruses, VLPs are entirely SARS-CoV-2 particles, consisting of the membrane protein (M), nucleoprotein (N), envelope protein (E), and S protein. Moreover, VLPs are likely a more faithful representation of SARS-CoV-2 S processing than pseudovirions because VLPs acquire their S protein from the endoplasmic reticulum (ER)-Golgi Intermediate Compartment (ERGIC) [38,39]. We observed that both WT and the ΔMBS mutant S proteins incorporated into SARS-CoV-2-VLPs had undergone cleavage, with almost complete processing of the WT protein and a minor fraction of the ΔMBS cleaved (Fig 2D). Processing of VLP-incorporated S protein was furin-dependent, as the proportion of cleaved S of either WT or ΔMBS mutant was reduced in 293T-ΔFURIN cells (Fig 2D). The cleavage of S protein incorporated in GSAS VLPs was completely abolished both for 293T and ΔFURIN cells, suggesting that removal of all basic residues is required to fully prevent S cleavage. It also supports the hypothesis from the pseudovirus data that evolution of the multibasic site has substantially enhanced the existing S cleavage at this position, which is mediated by the single arginine site. Importantly, the VLP data shows that furin is not essential for cleavage at the S1/S2 domain boundary, but the presence of an arginine residue is, suggesting that processing can be carried out by other cellular proteases. Having a multibasic sequence increases S1/S2 processing, with a single arginine residue being the minimal requirement.

As a final confirmation of these results, we examined S processing in SARS-CoV-2 wild-type virus. We infected 293T-hACE2 and 293T-hACE2-ΔFURIN cell lines and collected produced virus after 42 hours. The pattern of S-cleavage observed was similar to that seen for pseudoviruses and VLPs (Fig 2E), confirming that furin carries out, but is not absolutely essential, for S1/S2 processing. Moreover, this data proves the suitability of pseudoviruses and VLPs as surrogate models to study S protein cleavage.

### Processing of the S protein multibasic site is essential for cell-cell fusion

**Fig 3:**
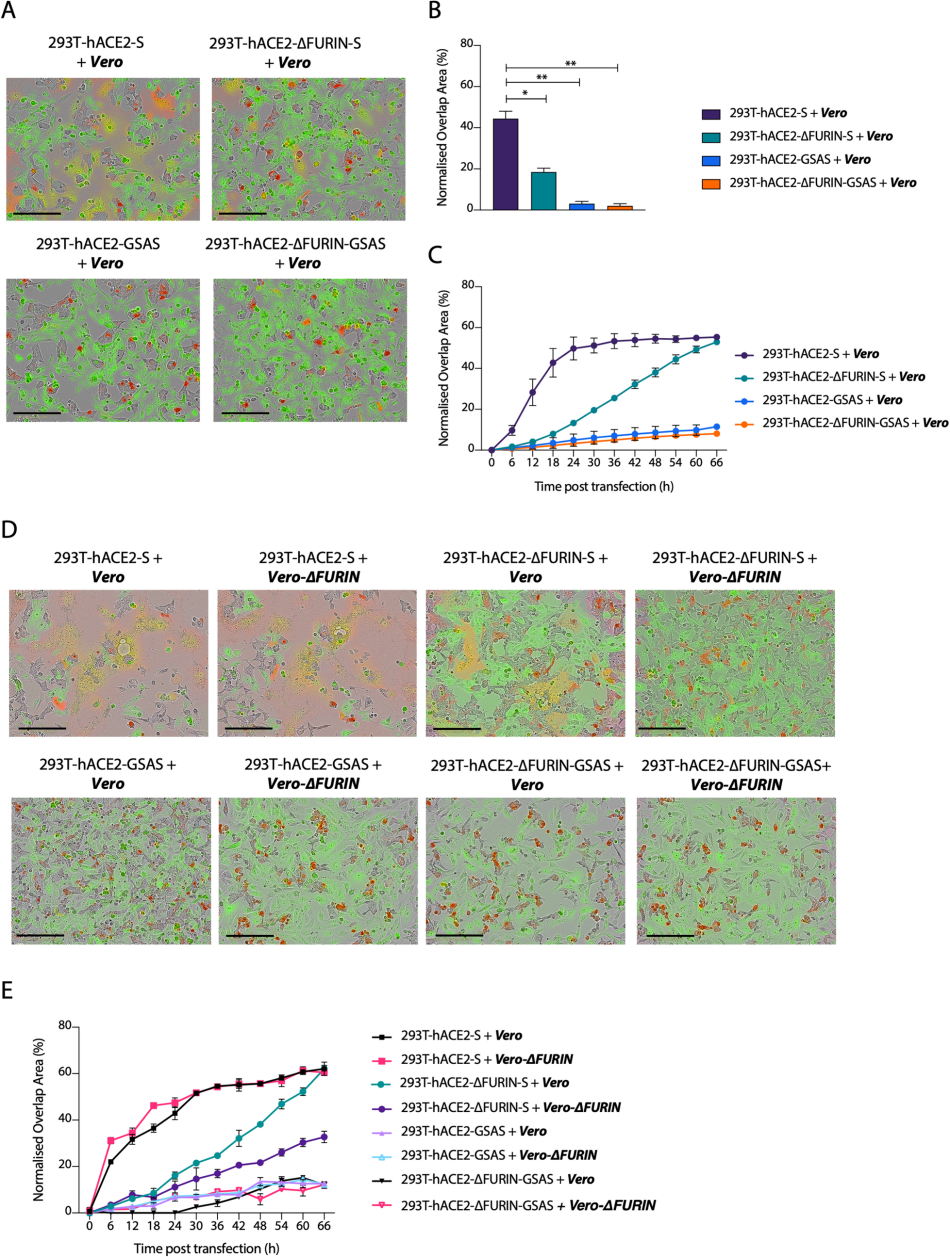
Processing of the S protein multibasic site is essential for cell-cell fusion but furin is dispensable. **(A)** and **(D)** Reconstructed images of the indicated cells lines transfected with the indicated S mutants and mixed with green-labelled cells at 24 hours post transfection. Scale bar 200 μm. Green colour identifies the acceptor cells while red colour marks donor cells. Merged green-red colours indicate the syncytia. **(B)** Quantification of green-red overlap area shown in (A) *P<0.05; **, *P<*0.01 analysed using Student *t*-test. **(C)** Time course of cell-cell fusion shown in (A). **(E)** Cell-cell fusion time course of images shown in (D). Data are expressed as mean +/- SEM (n = 2). Acceptor cells are marked in **bold** and *italics*.

Having determined the requirement for furin in S processing on virions, we sought to investigate how S cleavage at the multibasic site impacts the formation of multinucleated cells. To test this, we overexpressed SARS-CoV-2 S in 293T-ΔFURIN or 293T parental cells and measured cell-cell fusion activity. Overexpression of S in 293T-ΔFURIN cells still triggered syncytia formation when mixed with Vero cells (Fig 3A and 3B), albeit with slower kinetics compared to parental 293T cells (Fig 3C). This result is consistent with the idea that reduced SARS-CoV-2 S cleavage in 293T-ΔFURIN cells results in fewer cell-cell fusion events. To rule out the possibility that furin from Vero cells could cleave S produced in 293T-ΔFURIN cells, we generated a CRISPR-derived Vero cell line knocked out for furin (Vero-ΔFURIN) (S2A Fig). Mixing the two ΔFURIN cell types, we observed that the absence of furin in acceptor cells further decreased cell-cell fusion kinetics (Fig 3D and 3E), but formation of multinucleated cells still occurred (Figs 3E and S2B), suggesting that furin from the acceptor cells contributes to syncytia formation but it is not a major determinant. Overexpression of the uncleavable GSAS mutant did not induce cell-cell fusion in any of the above-mentioned conditions, suggesting that whilst furin itself is not essential, cleavage at the multibasic site is a requirement for syncytia formation (Fig 3A and 3E). These data together suggest that in the absence of furin, S protein can still be cleaved by another protease that recognises the multibasic site and is able to induce the formation of multinucleated cells.

### Furin ablation decreases SARS-CoV-2 replication but increases S-pseudovirus entry

**Fig 4:**
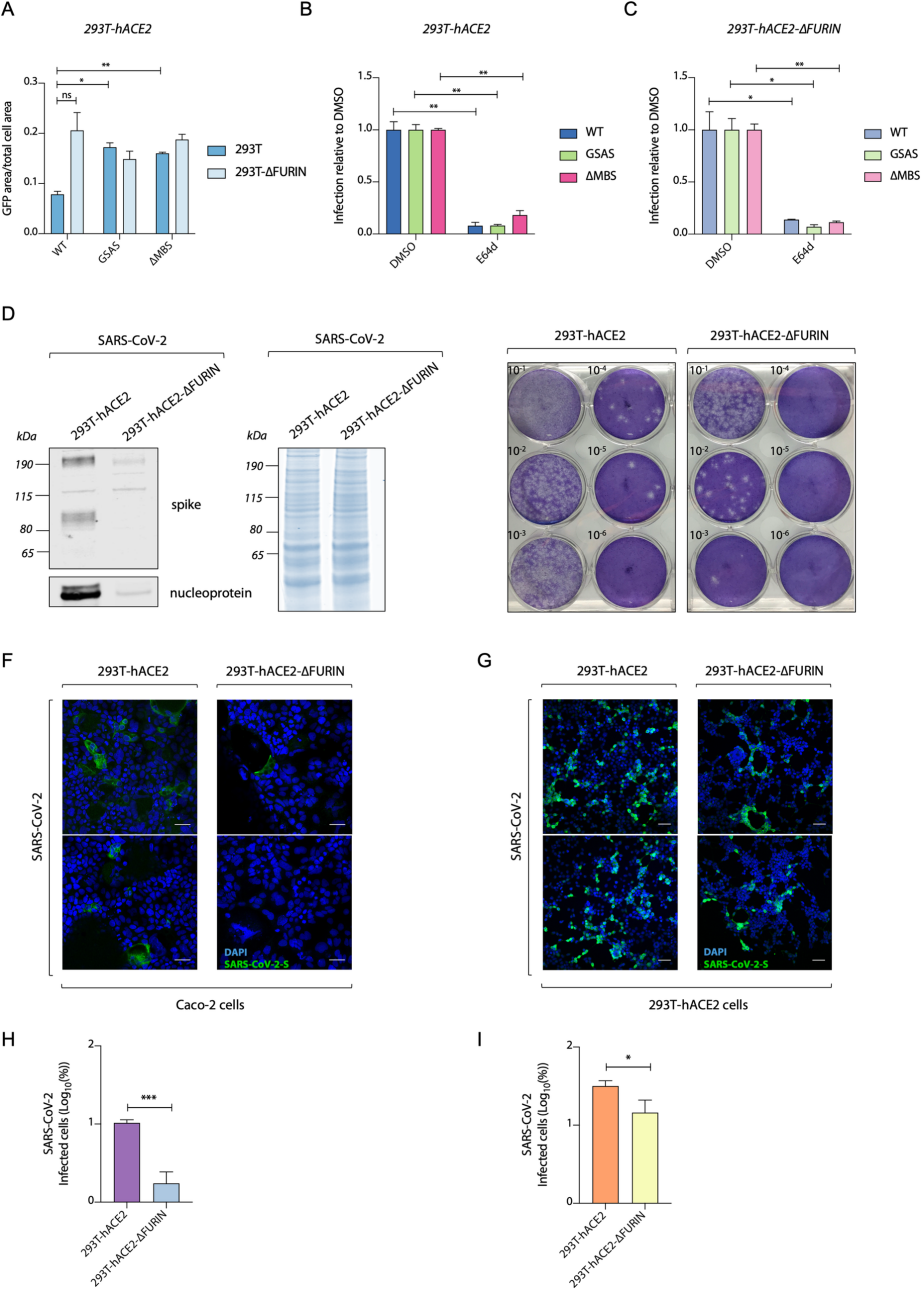
Furin enhances SARS-CoV-2 replication but is not essential for S-pseudovirus entry. **(A)** Infection of 293T-hACE2 cells with GFP expressing HIV pseudotyped with SARS-CoV-2 S mutants, measured as proportion of cell area expressing GFP. Viruses were produced in either 293T-wt or 293T-ΔFURIN cells. **(B)** and **(C)** Infection data of 293T-hACE2 cells with HIV pseudotyped with SARS-CoV-2 S mutants as in (A), with cells pre-treated for 2 hours with either DMSO or 25μM lysosomal inhibitor E64d as indicated. Statistical analysis was performed using Student *t*-test *P<0.05; **P<0.01, “ns” not significant. **(D)** Representative western blot of viral particles produced from SARS-CoV-2 infection of 293T-hACE2 and 293T-hACE2-ΔFURIN cells at 72 hours post infection. Spike and nucleoprotein are detected (left panel). Total protein content of virus preparation by Coomassie staining (right panel). **(E)** Plaque assay of Vero-hACE2-TMPRSS2 infected with viruses produced in D. **(F)** and **(G)** Immunofluorescence images displaying infection of Caco2 BVDV-Npro cells (F) and 293T-hACE2 (G) with equalised amounts of SARS-CoV-2 virus produced in 293T-hACE2 and 293T-hACE2-ΔFURIN cells. Spike (green) and nuclei (blue) are shown. Scale bar, 50 μm. **(H)** and **(I)** Quantification of SARS-CoV-2 infected cells shown in (F) and (G). *P<0.05; ***, *P<*0.001 analysed using Student *t*-test. Data are expressed as mean +/- SEM (n = 2).

Having assessed the role of S cleavage in triggering cell-cell fusion, we investigated how furin processing of SARS-CoV-2 S influences the infectivity of viral particles. To test this, we produced both WT and multibasic site mutant SARS-CoV-2 S pseudoviruses in 293T-ΔFURIN cells or 293T parental cells and infected 293T-hACE2 cells. Pseudoviruses harbouring wild-type S were less infectious when produced in 293T cells compared to viruses produced in 293T-ΔFURIN. This difference was lost when using pseudovirions expressing either the GSAS or ΔMBS mutant S, which showed increased infection compared to those expressing WT S, consistent with the fact that furin acts on the multibasic site (Fig 4A). Many coronaviruses, including SARS-CoV and SARS-CoV-2 exploit endocytosis for entry into 293T cells but the role of S cleavage in orchestrating this mechanism is not completely understood. To investigate how S processing can impact on the endocytic viral entry route, we produced pseudoviruses in 293T-ΔFURIN or 293T parental cells and compared infection of 293T-hACE2 cells in the presence of E64d, a widely used inhibitor of lysosomal cysteine proteases. We found that S-pseudovirions produced in both ΔFURIN and parental 293T cells showed a strongly impaired entry in the presence of E64d (Fig 4B and 4C). Together these data indicate that protease-mediated S processing at the multibasic site is not required for infection of 293T cells, and that the virus is able to enter via an endocytic mechanism regardless of any S pre-cleavage that has occurred in producer cells. MERS-CoV infection of 293T cells can occur via cleavage at the S2’ site by cathepsin in late endosomes [40].

Having determined that pre-cleavage of S at the multibasic site in the producer cells is not essential for pseudovirus entry, we sought to investigate the role of furin in SARS-CoV-2 wild-type virus replication. We infected cells with MOI of 0.01 and left viruses to spread for 72 hours. Western blot analysis showed that fewer viral particles were produced in 293T-hACE2-ΔFURIN cells than in 293T-hACE2 parental cells, as indicated by the significantly lower amount of nucleoprotein (Fig 4D, left panel), but equal total protein content (Fig 4D, right panel). Viral titration and plaque assay in Vero-hACE2-TMPRSS2 cells confirmed this result and showed that loss of furin leads to a reduction in the production of infectious virus by two orders of magnitude (Fig 4E). To test whether depletion of furin reduces virion infectivity rather than numbers of virions, we normalised the amount of virus produced in 293T-ΔFURIN and parental cells by nucleoprotein expression and infected Caco2 or 293T-hACE2 cells, which are cells routinely used for SARS-CoV-2 infection studies [41]. Using immunofluorescence of S protein to detect infected cells at 24 hours post infection, we observed that virions produced in 293T-hACE2-ΔFURIN cells were markedly less infectious in Caco2 cells (Fig 4F and 4H), while this difference was less evident in 293T-hACE2 cells (Fig 4G and 4I). The reduction in Caco2 infection by viruses produced in furin KO cells is consistent with the role of furin pre-cleavage in making the S2’ site accessible for TMPRSS2. TMPRSS2 is expressed on the surface of Caco2 cells, where it promotes SARS-CoV-2 entry by virus-cell membrane fusion [4]. Conversely, in 293T cells, SARS-CoV-2 can enter by an endosomal pathway [14], where other endosome-associated proteases are able to trigger membrane fusion regardless of S pre-cleavage. This result is consistent with the hypothesis that pre-cleavage of S protein by furin in producer cells at the S1/S2 boundary is required in order for TMPRSS2 to access and cleave the S2’ domain and trigger virus-cell membrane fusion. Thus, furin can contribute to virus spread by increasing virion infectivity.

### TMPRSS2 protease is required on acceptor cells to trigger cell-cell fusion

**Fig 5:**
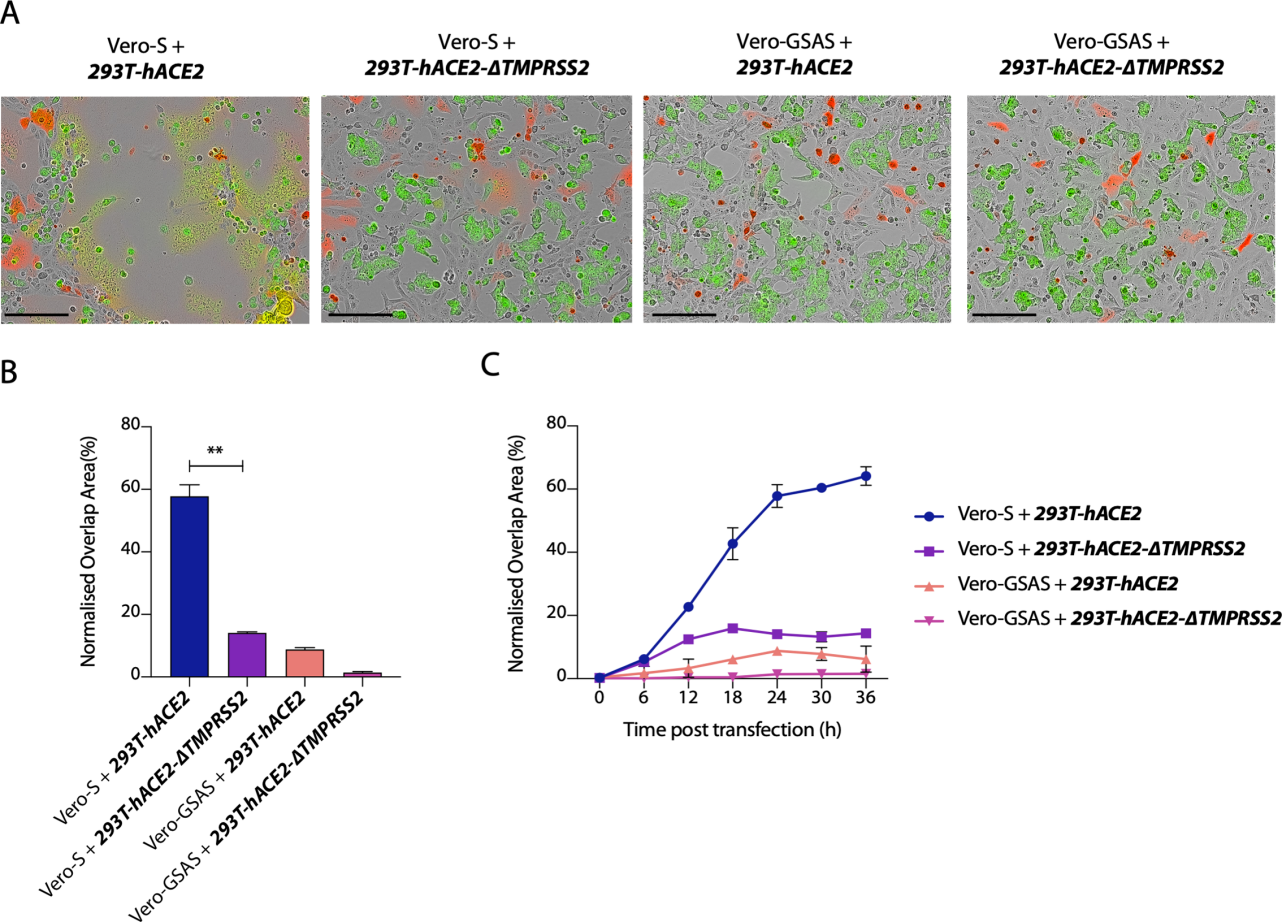
TMPRSS2 protease is required on acceptor cells to trigger cell-cell fusion. **(A)** Merged images of the indicated cells lines expressing the indicated S mutants and mixed with green-labelled cells at 24 hours post transfection. Scale bar 200 μm. Green colour identifies the acceptor cells while red colour marks donor cells. Merged red-green colours indicate the syncytia. **(B)** Quantification of percentage of green-red overlap area shown in (A) at 24 hours post transfection. Statistical analysis was performed using Student *t* test. **P<0.01. **(C)** Time course of cell-cell fusion as in (B). Data are expressed as mean +/- SEM (n = 2). Acceptor cells are marked in **bold** and *italics*.

It is thought that TMPRSS2 cleavage of the S2’ domain triggers virus-plasma membrane mixing by exposing the fusion peptide. Whether TMPRSS2 protease is similarly required for cell-cell fusion and if this favours SARS-CoV-2 virus spreading among cells, is incompletely understood. We tackled this question with our cell-cell fusion assay, using CRISPR-derived 293Ts knocked out for TMPRSS2 (293T-ΔTMPRSS2) as the acceptor cells (S3A and S3B Fig). The absence of TMPRSS2 protease on the acceptor cell prevented the formation of syncytia (Fig 5A and 5C). A previous report suggested hACE2 can be processed by TMPRSS2 [42], however we observed no hACE2 cleavage upon TMPRSS2 over-expression (S1A Fig). This suggests that TMPRSS2 processing of S at the S2’ site is important in triggering cell-cell fusion. The GSAS mutant failed to induce multinucleated cells (Fig 5A and 5C), consistent with the hypothesis that S pre-cleavage at the multibasic site is necessary to facilitate TMPRSS2-mediated processing at the S2’ site to expose the fusion peptide and trigger fusion between cell membranes.

## Discussion

One of the key distinguishing features between SARS-CoV-2 and SARS-CoV is the presence of a multibasic site comprising several arginine residues (R) between the S1/S2 domains of the major surface glycoprotein S [9,43]. The multibasic site has been shown to result in the cleavage of SARS-CoV-2 S [13]. The protease responsible for this activity is thought to be furin but this conclusion is largely based on experiments using small molecule inhibitors [3,26,36]. Here we show that genetic knockout of furin significantly reduces but does not abolish SARS-CoV-2 S processing. This indicates that the multibasic site is also a substrate for other proprotein convertases. One candidate protease that can play a role in this process is the serine protease matriptase, which also prefers sequences with R-X-X-R amino acid sequence and has been shown to activate the hemagglutinin protein of H9N2 influenza A viruses that have the cleavage site motif R-S-S-R [44,45]. In the case of SARS-CoV-2 S, we found that the presence of only one R residue between the S1/S2 subunits significantly reduces cleavage efficiency and this is further reduced, but not abolished, in furin-deficient cell lines.

Importantly, our data show that furin cleavage strongly promotes SARS-CoV-2 replication. Substantially fewer virions were produced in furin knockout cells at 72 hours post infection. Plaque assays confirmed this result and showed that loss of furin decreased the production of infectious virions by two orders of magnitude. This was due to a loss of virion infectivity rather than a reduction in viral production, as when equal numbers of virions were used to infect Caco2 cells, we saw a significant reduction in S protein expression, marker of viral replication, over 24 hours. Immunoblot analysis of viruses produced in furin knockout cells showed that S protein was no longer efficiently cleaved. Taken together, this data shows that furin pre-processes S protein and promotes the subsequent infection of Caco2 cells, while only slightly affecting the entry in 293T cells. This is consistent with previous data suggesting that SARS-CoV-2 infects Caco2 cells via plasma membrane fusion and that pre-processing of S is required in order for TMPRSS2 to cleave S2’ and expose the fusion peptide for membrane mixing [4,26]. Conversely, in 293T cells, the virus enters predominantly via an endosomal pathway in which endosome-associated proteases are able to process S at both the S1/S2 and S2’ sites to drive virus-endosome membrane fusion. Data from S-pseudotyped virions was similar to that of SARS-CoV-2 in that loss of furin did not prevent 293T infection, although there was a small increase rather than decrease in overall levels. This suggests that S-pseudovirions favour endosomal entry when infecting 293Ts slightly more than SARS-CoV-2.

Furin cleavage and the multibasic site is also thought to be important for the ability of Coronavirus S protein to mediate cell-cell fusion [12,36,46]. This is potentially significant for SARS-CoV-2 virulence as it provides an additional route for disseminating virus through the host. The ability to transmit between cells in a particle-independent manner also has implications for pathogenesis, as we speculate that immune factors, such as antibodies, may be less able to block this type of spread. We therefore investigated whether furin is required for cell-cell fusion and the formation of multi-nucleated syncytia. Unlike with pseudovirus infection of 293T cells, we find that deletion of furin from donor 293T cells decreases S-mediated cell-cell fusion with nearby acceptor cells. Unexpectedly however, we find that while loss of furin leads to a slowing of cell-cell fusion, it does not prevent it. Furthermore, we still observed syncytia formation when furin was deleted from both donor and acceptor cells. In contrast, complete removal of the multibasic site abolished S-mediated fusion. This suggests that while furin is the main protease involved in pre-processing S protein for cell-cell fusion, other proteases can cleave at the S1-S2 junction. Thus, while blocking cleavage of S1-S2 is likely to halt this mode of viral transmission, inhibitors may need to block more than just furin for complete efficacy.

A clear difference between our cell-cell fusion and pseudovirus experiments was that while pseudovirus infection and processing data are the same for the GSAS mutant and ΔFURIN cells, there is a substantial variation in cell-cell spread between the two perturbations. As cell-cell fusion provided a more accurate model for furin-dependence, we used this assay to test other cofactor and cell dependencies. We found that S protein mediated cell-cell fusion was possible between different cell types, with no requirement for a particular cell type as donor or acceptor. We also found that manipulation of 293T cells greatly altered their ability to participate in fusion as overexpression of hACE2 or TMPRSS2 greatly increased syncytia formation. Conversely, knockout of TMPRSS2 from 293Ts cells abolished fusion. Fusion could not be restored when only donor cells expressed TMPRSS2. This shows that the SARS-CoV-2 S protein feature of forming multinucleated cells is dependent on the spatial orientation of TMPRSS2 in relation to the S protein. TMPRSS2 must be present in the opposing cell membrane to activate S protein and induce cell-cell fusion. This has important implications for predicting which cell types and tissues are susceptible for cell-cell viral spread: TMPRSS2 and ACE2 are essential but furin is not. Despite the rapid development of effective vaccines against SARS-CoV-2, antiviral drugs and specific inhibitors of enzymes involved in viral replication are urgently needed. Our findings indicate that, since furin is not essential for virus replication, inhibitors currently used against this cellular protease may reduce but not completely block viral infection. This study also highlights the targeting of cell-cell fusion process as a potential antiviral strategy, fostering the development of specific inhibitors able to limit the spread of SARS-CoV-2.

## Materials and methods

### Cells

HEK293T CRL-3216, Vero CCL-81 were purchased from ATCC and maintained in Dulbecco’s Modified Eagle Medium (DMEM) supplemented with 10% fetal calf serum (FCS), 100 U/ml penicillin, and 100mg/ml streptomycin. All cells are regularly tested and are mycoplasma free.

Caco2 BVDV-Npro cell line was generated as described previously [47].

293T-hACE2, 293T-hACE2-ΔFURIN, Vero-hACE2 were generated transducing 293T, 293T-ΔFURIN or Vero cells with lentiviral particles expressing hACE2 ORF and cultured in DMEM 10% FCS with 5 μg/ml blasticidin. 293T-hACE2-TMPRSS2 and Vero-hACE2-TMPRSS2 were generated by transducing 293T-hACE2 and Vero-hACE2 cells respectively with lentiviral particles expressing TMPRSS2 ORF and maintained in DMEM 10% FCS with addition of 5 μg/ml blasticidin and 1 μg/ml puromycin.

### Viruses

The SARS-CoV-2 virus used in this study is the clinical isolate named "SARS-CoV-2/human/Liverpool/REMRQ0001/2020”, isolated by Lance Turtle (University of Liverpool) and David Matthews and Andrew Davidson (University of Bristol).

### Plasmids

Vectors for viral production, were, for HIV-1 Gag Pol, pCRV-1 [48] and for GFP expression, CSGW [49].

For production of VLPs codon optimised plasmids encoding M,N,E,S protein were generated using DNA gblocks by IDT. The gBlocks were cloned into pcDNA3 using HindIII and XhoI restriction enzyme sites. pCAGGS-S used for cell-cell fusion assay was generated cloning codon optimised S into a pCAGGS empty backbone using EcoRI and NheI restriction sites.

pCAGGS-S Δc19 used for pseudotyping lentiviruses was generated deleting the last 19 amino acids, from the cytoplasmic tail of the S protein, which have been previously shown to contain an endoplasmic reticulum (ER)-retention signal [39].

pCAGGS-GSAS was obtained by using QuikChange II Site-Directed Mutagenesis (Agilent Technologies). The same strategy was used to eliminate PRRA residues from pCAGGS-S harbouring the pCAGGS-ΔMBS.

The plasmids used for CRISPR-Cas9 gene editing were pSpCas9(BB)-2A-GFP (PX458, Addgene ID 48138) and pSpCas9(BB)-2A-Puro (PX459, Addgene ID 48139). pX458 and pX459 were digested with BbsI and dephosphorylated with FastAP Thermosensitive Alkaline Phosphatase. Primers containing gRNA sequences were annealed and phosphorylated by T4 Polynucleotide Kinase and the annealed oligonucleotides were inserted into the pX458 or pX459 vector by ligation. The following gRNA sequences were used: 5′ -GATGCGCACAGCCCACGTGT- 3′ targeting human FURIN (in pX459, pJC142), 5′ -CGGATGCACCTCGTAGACAG- 3′ and 5′ -TGTGCCAAAGCTTACAGACC- 3′ targeting human TMPRSS2 (in pX459, pJC144 and pJC146 respectively) and 5′ -ATGCTTCCGTGCCACGCTAT- 3′, 5′ -GCTGAGGTCCTGGTTGCTAT- 3′ and 5′ -CGGTGCTATAGTGTGTATCG- 3′ targeting African green monkey FURIN (in pX458, pJC228, pJC229 and pJC232).

### Virus stock

SARS-CoV-2 stock was prepared in Vero hACE2-TMPRSS2 cells. Cells were infected with 0.01 MOI of virus and incubated for 3 days. Virus stock was harvested by three freeze-thaw cycles followed by 5 min 300xg spin to remove cell debris. Titres were assessed by plaque assays in Vero hACE2/TMPRSS2 cells.

### Preparation of S pseudotyped HIV-1 virions

Replication deficient VSV-G or SARS SARS-CoV-2 pseudotyped HIV-1 virions were produced in 293T wild-type (293T-wt) cells by transfection with pMDG2 or pCAGGS-S Δc19, pCRV GagPol and CSGW as described previously [50]. Viral supernatants were filtered through a 0.45μm syringe filter at 48 hours post-transfection and pelleted through a 20% sucrose cushion for 2hrs at 90000 g. Pelleted virions were drained and then resuspended in OptiMem.

### VLP production and S analysis

SARS-CoV-2 VLPs were produced by transfecting 2x10^6^ 293T-wt cells in T75 flasks with 25 μg total DNA using Fugene6 (Promega). The codon optimised plasmids expressing SARS-CoV-2 M, N, E, and S proteins were transfected in a ratio of 1:5:5:1. VLPs were harvested 48 hours post transfection from the culture supernatants, cleared by 0.45 μm filtration, and partially purified by pelleting through a 20% sucrose layer for 2hours at 90000 g. VLP pellets were resuspended in 50 μl of DMEM, and the 20μl were loaded on the 4–12% Bis-Tris gel for WB analyses.

### Generation of hACE2 and TMPRSS2 lentiviral particles

For the generation of hACE2 over-expressing 293T cells, the human hACE2 ORF was PCR amplified from Addgene plasmid 1786 and C-terminally fused with the porcine teschovirus-1-derived P2A cleavage sequence (ATNFSLLKQAGDVEENPGP) followed by the blasticidin resistance gene. This continuous, single ORF expression cassette was transferred into pLenti6-Dest_neo by gateway recombination. The TMPRSS2 (gene bank accession number AF123453.1) ORF was synthesised and cloned into pLenti6-Dest_Puro by gateway recombination. Lentiviral particles were generated by co-transfection of 293T cells with pLenti6-Dest_neo_ACE2-2A-Bla or pLenti6-Dest_Puro_TMPRSS2 together with pCMVR8.74 (Addgene plasmid 22036) and pMD2.G (Addgene plasmid 12259) using PEI. Supernatant containing virus particles was harvested after 48 h, 0.45 um filtered, and used to infect cells to generate stable cell lines. Transduced cells stably expressing hACE2 or TMPRSS2 were selected with 5 μg/ml blasticidin and 1 μg/ml puromycin, respectively.

### Generation of 293T-ΔFURIN and 293T-ΔTMPRSS2

293T cells were grown to ~70% confluence and transfected with a mixture of 2 μg of DNA encoding the gRNA and 6 μL of Polyethylenimine (PEI, Polysciences catalogue #24765, diluted to 1 mg/mL in PBS) in 100 μL Opti-MEM. Cells were transfected with 2 μg of pJC142 to knock-out *FURIN* or co-transfected with 1 μg of pJC144 and 1 μg of pJC146 to knock-out *TMPRSS2*. 48 hours later, the cells were trypsinized and replated in complete medium containing 1.5 μg/mL puromycin. 48 hours later (day 4), cells were diluted to 1 cell per two wells in 96-well plates and grown in non-selective complete medium. Single colonies that grew were expanded and analysed by immunoblotting for *ΔFURIN* clones and genotyping PCR for *ΔTMPRSS2* clones.

For the generation of Vero-*ΔFURIN* were co-transfected with the plasmids pJC228, pJC229 and pJC232 in which a mixture containing 0.66 μg of each plasmid and 6 μL of PEI in 100 μL opti-MEM was added to cells. 48 hours later, cells were trypsinized and single GFP positive cells were sorted into each well of 96-well plates using a Synergy 1 FACS sorter. Single colonies were expanded and analysed by Immunoblotting.

### S pseudotypes infection experiments

Cells were plated into 96 well plates at a density of 7.5x10^3^ cells per well and allowed to attach overnight. Viral stocks were titrated in triplicate by addition of virus onto cells. Infection was measured through GFP expression measured by visualisation on an Incucyte Live cell imaging system (Sartorius). Infection was enumerated as GFP positive cell area. For treatment with the inhibitor E-64d, cells were pre-treated with 25μM for 2 hours prior to addition of the virus.

### Cell-cell fusion assay

Acceptor cells and donor cells were seeded at 70% confluency in a 24 multiwell plate. Donor cells were co-transfected with 1.5 μg pCAGGS-S and 0,5 μg pmCherry-N1 using 6 μl of Fugene 6 following the manufacturer’s instructions (Promega). Acceptor cells were treated with CellTracker™ Green CMFDA (5-chloromethylfluorescein diacetate) (Thermo Scientific) for 30 minutes according to the manufacturer instructions. Donor cells were then detached 5 hours post transfection, mixed together with the green-labelled acceptor cells and plated in a 12 multiwell plate. Cell-cell fusion was measured using an Incucyte and determined as the proportion of merged area to green area over time. Data were then analysed using Incucyte software analysis. Data were normalised to cells transfected only with mCherry protein and mixed with green labelled acceptor cells. Graphs were generated using Prism 8 software.

### SARS-CoV-2 plaque assay

Vero hACE2-TMPRSS2 cells were seeded on 12-well plates day prior infection. Next day cells were infected using serial dilutions of the supernatant -1 to -6 for 1h and then overlayed with 0.05% agarose in 2% FBS DMEM. After 3 days cells were fixed with 4% formaldehyde and stained with 0.1% toluidine blue.

### Immunofluorescence

Immunofluorescence experiments were performed using μ-Slide 8 Well Chamber Slide-well (iBidi GmbH). Caco-2 cells or 293T-hACE2 cells were infected with SARS-CoV-2 virus produced in 293T-hACE2 or 293T-hACE2-ΔFURIN. In order to add approximately the same number of viral particles for both conditions, normalisation was performed by quantifying the SARS-CoV-2 nucleoprotein band in the Western blot (Fig 4D) using ImageJ software. Cells were fixed with 4% formaldehyde after 24 hours post infection, permeabilised with 0.1% Triton X-100 and then blocked with 5% FBS. Primary antibody used was mouse anti-spike (GeneTech, clone 1A9) and secondary was anti-Mouse Alexa Fluor 488. Nuclei were stained with Hoechst 34222. Images were taken with Zeiss 710 confocal microscope. Image processing was performed using ImageJ software.

### Virus infection

293T-hACE2 or 293T-hACE2-ΔFURIN cells were infected with MOI of 0.1 or 0.01 and incubated for 42 hours or 72 hours depending on the experiment. To concentrate released virions supernatant was incubated with 10% PEG6000 (4h at RT) and then pelleted by 30min spin at 12000 g. Pellets were resuspended directly in Laemmli buffer with 1mM DTT. Cells were lysed with Laemmli buffer with 1mM DTT and then treated with Benzonase Nuclease(70664 Millipore) and sonicated prior loading for gel electrophoresis.

### Genotyping PCR

293T-Δ*TMPRSS2* cells were screened by genotyping PCR. Genomic DNA was extracted from wild-type and Δ*TMPRSS2* 293T cell lines using the Puregene cell kit according to the manufacturer’s instructions (Qiagen, #158388). Co-transfection of pJC144 (guide 1) and pJC146 (guide 2) was expected to result in the deletion of a large part of the *TMPRSS2* gene and in order to screen for large deletions, a pair of primers labelled as primers 1 and 3 (oJC545 5’ -GCCACCGCACCCAGCCTTGTAGTAC- 3’ and oJC554 5’ -TTCCAGCAGCAGAACCACGCC- 3’) were used for PCR amplification. For alleles in which large deletions had not occurred, smaller frameshift-inducing indels caused by guide 1 in the second exon were screened by PCR using the primer pair primer 1 and 2 (oJC545 and oJC552 5’ GCGACAGTGGTGTTGGGAGCAG 3’). All PCR reactions were conducted using Phusion polymerase (NEB) according to the manufacturer’s instructions with GC buffer, 1.2% DMSO, 2 ng/μl genomic DNA, an extension time of 16 seconds and an annealing temperature of 64°C. PCR products were resolved on 2.4% agarose gels with SYBR Safe (Thermo Fischer Scientific, #S33102) and relevant bands were excised from the gels, purified using the Qiagen gel extraction kit (#28704, used according to the manufacturer’s instructions) and sanger sequenced using primer 1 or 3. Alignments of sequence trace files were generated using Snapgene.

### Western blot

Cells lines were resuspended in culture medium and centrifuged at 300 x g for 5 minutes at 4°C. Cells were washed once in PBS and resuspended in 1x LDS Sample buffer with β mercaptoethanol or in lysis buffer containing 50 mM Tris HCl pH 7.4, 150 mM NaCl, 1 mM EDTA, 0.1% Triton X-100, 1 mM PMSF (Sigma) and 1x complete EDTA-free protease inhibitor cocktail (Roche). Cells were treated with Benzonase Nuclease (70664 Millipore) or were incubated on ice for a further 10 minutes prior to the clarification of the lysate at 16,100 x g for 10 minutes at 4°C respectively. Samples were then sonicated and incubated at 90°C for 5 minutes. Samples were then run on 4%–12% Bis Tris gels and transferred onto nitrocellulose membranes using an iBlot (Life Technologies).

Membranes were blocked for 1 hour in 5% non-fat milk in PBS + 0.1% Tween-20 (PBST) at room temperature with agitation, incubated in primary antibody diluted in 5% non-fat milk in PBST overnight at 4°C with agitation, washed four times in PBST for 5 minutes at room temperature with agitation and incubated in secondary antibody diluted in 5% non-fat milk in PBST for 1 hour with agitation at room temperature. Membranes were washed four times in PBST for 5 minutes at room temperature with agitation and imaged directly using a ChemiDoc MP imaging system (Bio-Rad). Alternatively, blots probed with an anti-Alexa Fluor 488 secondary antibody were imaged using a Typhoon biomolecular imager (Cytiva).

The primary antibodies used were: anti-furin (1:1000, Abcam #ab3467); anti-α tubulin (1:250, YL1/2); anti-SARS-CoV-2 S, which detects the S2 subunit of SARS-CoV-2 S (Invitrogen, PA1-41165); anti-SARS-CoV-2 Nucleoprotein (ABIN129544); Anti-HIV p24 (183-H12-5C), obtained from the NIH AIDS Reagent Program; anti-TMPRSS2 (Clone P5H9-A3), Anti-hACE2 (EPR4435). The secondary antibodies were anti-rabbit HRP conjugate (1:10000, Invitrogen 31462), anti-β actin HRP (1:5000; sc-47778), anti-rat Alexa Fluor 488 (1:3000; Thermo Fischer Scientific A-21208).

## Supporting information

S1 Fig Generation of stable hACE2 and TMPRSS2 overexpressing 293T cell lines and deletion of FURIN in 293T cells by CRISPR-Cas9 gene editing. **(A)** and **(B)** Western blots showing hACE2 (A) and TMPRSS2 (B) levels in the indicated cell lines. β-actin was used as loading control. **(C)** Western blot of furin levels in 293T-ΔFURIN cells and 293T cells. Tubulin was used as a loading control. Immunoblots were repeated in duplicate.
(TIF)

S2 Fig Deletion of FURIN in Vero cells did not impact the formation of multinucleated cells. **(A)** Western blot showing furin levels in Vero and Vero-ΔFURIN cells. β-actin was used as a loading control. **(B)** Reconstituted images of the indicated cells lines transfected with WT S and mixed with green-labelled cells at 60 hours post transfection. Black arrows indicate multinucleated cells. Acceptor cells are marked in **bold** and *italics*.
(TIF)

S3 Fig Deletion of TMPRSS2 in 293T cells by CRISPR-Cas9 gene editing. **(A)** A schematic illustrating the strategies for the deletion of TMPRSS2 and for screening deletion clones by genotyping PCR. Blue rectangles (exons), black lines (introns), scale bar = 1000 bp. Guide 1 was designed to target an early constitutive exon while Guide 2 targeted a region near the 5’UTR so as to remove a large region of the open reading frame and/or cause early frameshift-inducing indels. A pair of primers 1 and 2 was used to screen for indels by sanger sequencing (see inset) while a pair of primers 1 and 3 was used to screen for large genomic deletions by agarose gel resolution **(B)** Genotyping PCRs were conducted in duplicate.
(TIF)
