# Supplementary figures and images for: Furin cleavage of SARS-CoV-2 Spike promotes but is not essential for infection and cell-cell fusion
Source: PLoS Pathog. 2021 Jan 25;17(1):e1009246. doi: 10.1371/journal.ppat.1009246 (PMC7861537; doi:10.1371/journal.ppat.1009246)

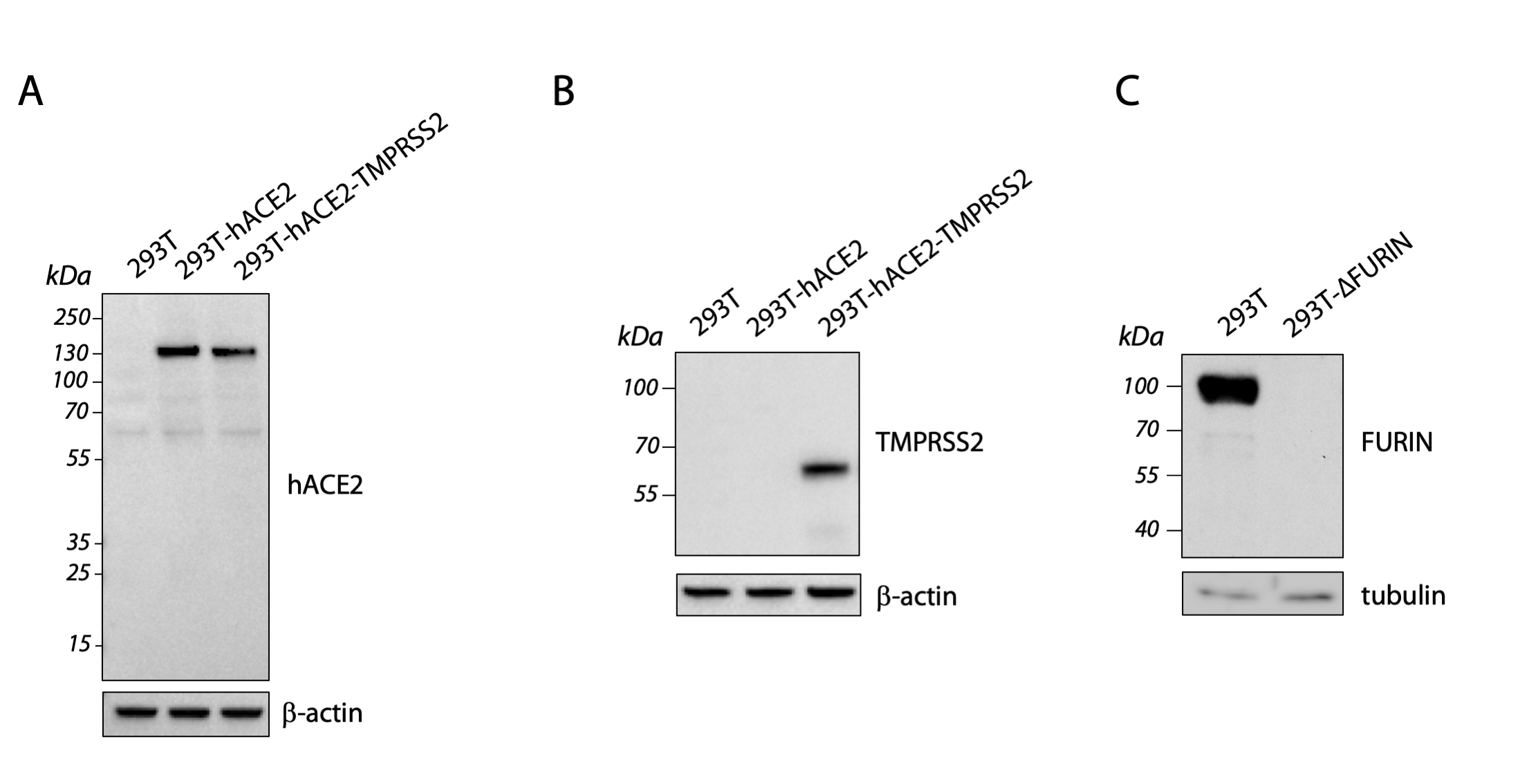

Supplement: S1 Fig — (A) and (B) Western blots showing hACE2 (A) and TMPRSS2 (B) levels in the indicated cell lines. β-actin was used as loading control. (C) Western blot of furin levels in 293T-ΔFURIN cells and 293T cells. Tubulin was used as a loading control. Immunoblots were repeated in duplicate. (TIF) [file ppat.1009246.s001.tif]

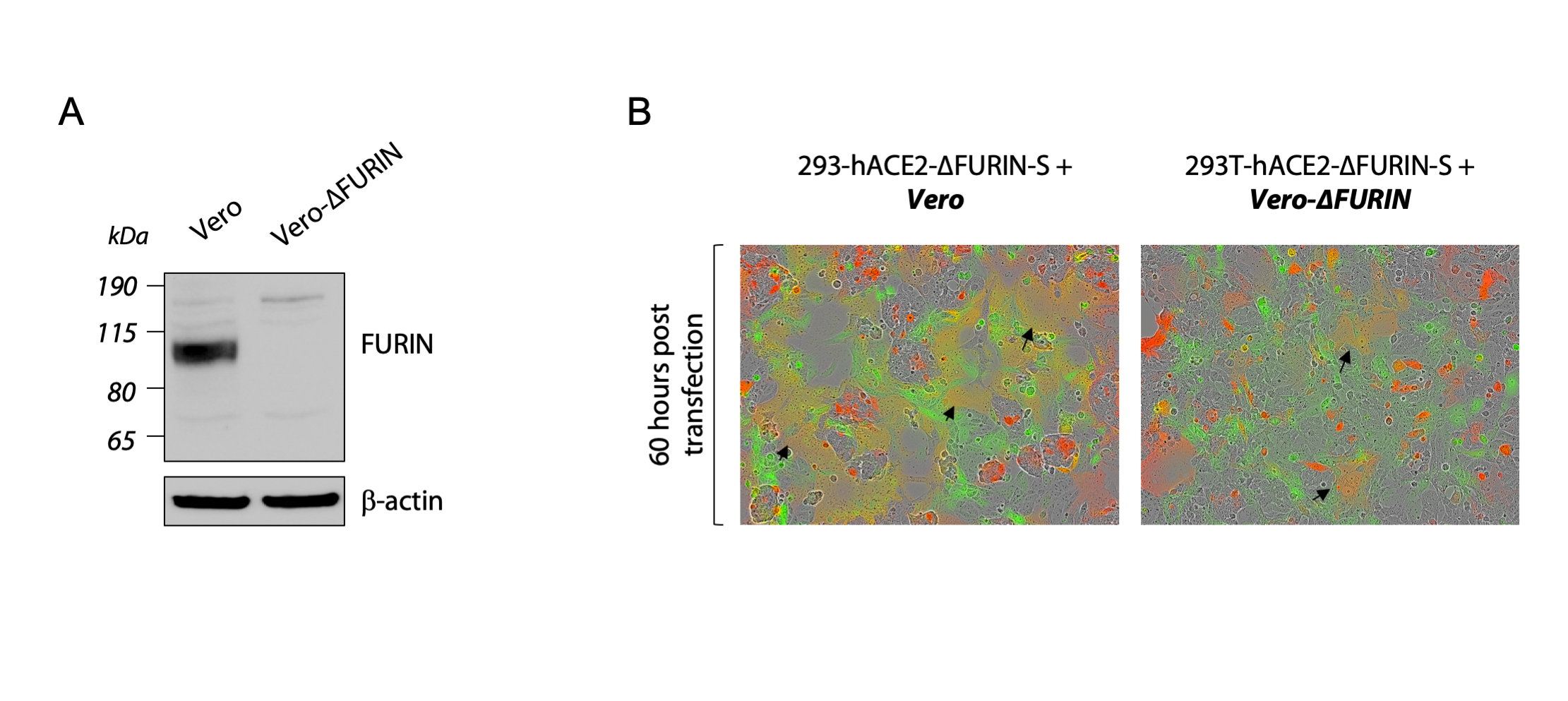

Supplement: S2 Fig — (A) Western blot showing furin levels in Vero and Vero-ΔFURIN cells. β-actin was used as a loading control. (B) Reconstituted images of the indicated cells lines transfected with WT S and mixed with green-labelled cells at 60 hours post transfection. Black arrows indicate multinucleated cells. Acceptor cells are marked in bold and italics. (TIF) [file ppat.1009246.s002.tif]

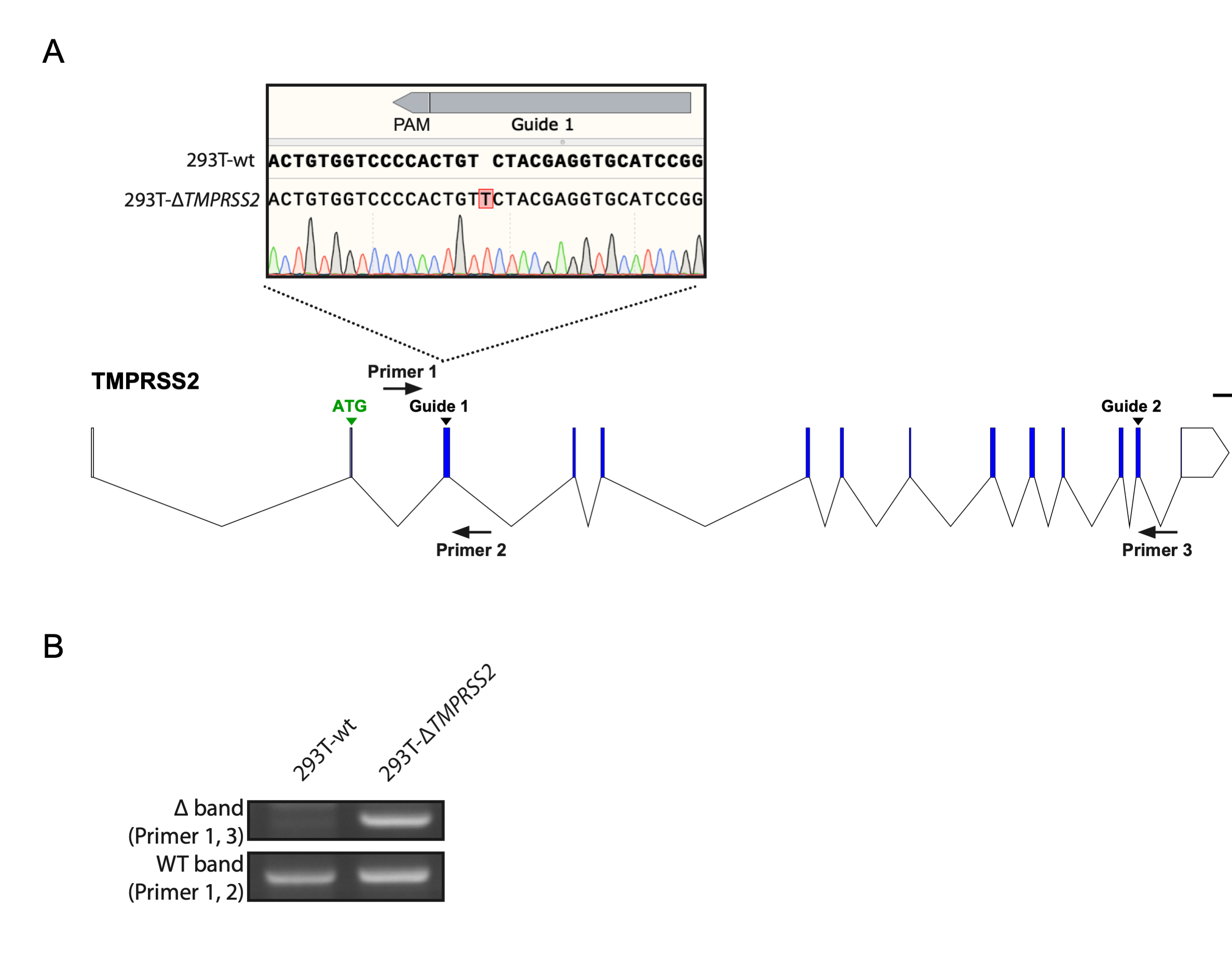

Supplement: S3 Fig — (A) A schematic illustrating the strategies for the deletion of TMPRSS2 and for screening deletion clones by genotyping PCR. Blue rectangles (exons), black lines (introns), scale bar = 1000 bp. Guide 1 was designed to target an early constitutive exon while Guide 2 targeted a region near the 5’UTR so as to remove a large region of the open reading frame and/or cause early frameshift-inducing indels. A pair of primers 1 and 2 was used to screen for indels by sanger sequencing (see inset) while a pair of primers 1 and 3 was used to screen for large genomic deletions by agarose gel resolution (B) Genotyping PCRs were conducted in duplicate. (TIF) [file ppat.1009246.s003.tif]
